# Institutional Notes and News

**Published:** 1909-12-25

**Authors:** 


### Institutional Notes and News.

**The Lambert Memorial.**

The 19th annual report of the Lambert Memorial Hospital has just been issued and shows that this little institution, which was founded by the late Mrs. Lambert for the benefit of the Thirsk and Sowerby district, is making good progress. The cost per patient per day has decreased from 5s. 8½d. in 1906 to 4s. last year, and the financial affairs of the institution are in a satisfactory condition. An interesting and detailed list of cases is appended to the report, giving the length of stay in hospital for each case. In some cases this period appears to have been very much above the average. Thus a case of "neurasthenia and dyspepsia" remained for 83 days, one of gastric ulcer 133, and one of osteo myelitis 371 days in hospital.

**The Hospital for Women, Soho Square, W.**

This hospital, founded in 1842, is the pioneer of women's hospitals. For many years past the necessity of rebuilding the institution has become more and more apparent, until delay became positively dangerous. The work, estimated to cost £20,000, was therefore taken in hand in March of this year, and the new hospital buildings will be ready for occupation in June 1910. The Rebuilding Fund now totals £12,000, so that £8,000 has still to be raised to complete the work. Of this latter sum King Edward's Hospital Fund have promised £3,000, on condition that the whole of the balance required to be raised is found by March 31, 1910. Here is an excellent opportunity for the charitable to assist a most worthy object this Christmas. It is earnestly to be hoped that the Institution will not lose this handsome conditional gift for want of the necessary money to fulfil the conditions of King Edward's Fund. Secretary, Mr. Alfred Hayward.

**Glasgow Mental Hospital.**

The medical superintendent's report for the past year is unusually interesting. It records the loss sustained by the institution through the death of Dr. Alex. Robertson, who had for 50 years been associated with lunacy work in Glasgow. Various structural alterations have been made. The balconies of the sick room have been roofed in, so as to render them more independent of wet weather. A small pavilion is in course of erection at the newly completed cricket ground. It is estimated to cost only £132 and will use up the stone and materials from the old weigh house, which is useless now that the weighs have been moved to the courtyard of the steward's store. To secure economy in the use of gravitation water and increase the efficiency of the stationary steam fire pumps, a new rainwater tank has been dug and almost completed. It will about double the rainwater storage and should correspondingly reduce the consumpt of gravitation water. On the upper terrace on the loch front of the female C and D blocks a new tennis court for the nursing staff has been laid out.

**An Experiment.**

In the same report mention is made of an important innovation which has been introduced with the object of endeavouring to re-educate the faculties of a large class of the patients who have lapsed into dementia, and who, in consequence, are unfit for any of the ordinary forms of employment. The experiment, limited at present to female patients, of the class described, and which has only been a few weeks in force, consists in training them to perform simple drill exercises, including musical drill and singing exercises, such as might be employed in schools for young children or for young persons of congenitally weak mind. For this purpose a qualified lady has been engaged, who also conducts singing classes in the various female wards, which are popular and appreciated by the patients. It is too soon to form an opinion regarding the effect of these exercises upon the debilitated mental faculties of many of the female patients, but the result will be watched with expectation, for there is no reason why it should not be attended with such a measure of success as will lead to a more extended trial. No systematic attempt on a large scale to attain this object has ever been made in this country before, and it is hoped that it may prove the commencement of a new movement.

**How the Money Goes.**

The Royal Hospital for Incurables, Putney, has issued a seasonable little book on the work and aims of the institution, which can serve in many respects as a model. It is a well-written, concise account of what is yearly done with the £35,000 that is expended on behalf of the hospital. By glancing over these thirty odd pages, anyone interested in the Hospital for Incurables can easily find out what is being done with the subscriptions, and get an insight, as far as it is possible to give such within the limits of a small booklet, into the work that is being done and the work that has still to be done. The pamphlet is well printed and got up, and illustrated with excellent views of the institution. It is worth while noticing that the hospital receives no grant from the King's, the Sunday, or the Saturday Funds, because it is not a hospital in the sense that it receives patients in order to cure them. To all intents and purposes it is a home and a refuge for those that cannot enter general or special hospitals, as their cases are considered incurable.

**Children's Ward for the Derbyshire Infirmary.**

The gift of Mr. A. Leslie Wright to the Derbyshire Royal Infirmary of a ward block exclusively for the use of children, at an estimated cost of about £8,000, completes the scheme for the new infirmary on the lines laid down and sanctioned by the Governors in 1891. This gift not only provides proper accommodation for children, but it also liberates beds in the existing wards for adult cases, for which they were originally intended, more particularly those properly belonging to the Gynæcological Department. The want of this accommodation has been most keenly felt by the Board and by the honorary medical staff in their work. This addition of thirty beds to the hospital will necessitate an increase on the present annual expenditure of the Infirmary of about £1,500.

**Extension at the Dundee Royal Infirmary.**

The Governors of the Dundee Royal Infirmary have sanctioned a scheme of extension, to cost £3,800, of which £2,400 has been already promised by the generosity of Mr. J. K. Caird, a famous benefactor of the town. The scheme will provide increased accommodation for the out-patient department and additional observation rooms for patients whom it is not considered desirable to place in the ordinary wards, besides additional new means of escape in case of fire in the servants' quarters. The two latter items are expected to cost £500 between them. The new out-patient department is justified by the fact that in the last ten years the number of out-patients at the hospital has increased 140 per cent. Various makeshifts have been employed meanwhile to meet this development, but the gift of Mr. Caird has now allowed a proper plan of reconstruction to be undertaken. The site which the new buildings will occupy is between the entrance gateways in Barrack Road—that is to say, on the west side of the infirmary. Mr. Finlay is to be the architect.

